# EUV stimulated emission from MgO pumped by FEL pulses

**DOI:** 10.1063/1.4993293

**Published:** 2017-08-10

**Authors:** Philippe Jonnard, Jean-Michel André, Karine Le Guen, Meiyi Wu, Emiliano Principi, Alberto Simoncig, Alessandro Gessini, Riccardo Mincigrucci, Claudio Masciovecchio, Olivier Peyrusse

**Affiliations:** 1 Laboratoire de Chimie Physique - Matière et Rayonnement, Sorbonne Universités, UPMC Univ. Paris 06, 4 place Jussieu, F-75252 Paris cedex 05, France; 2 CNRS UMR 7614, Laboratoire de Chimie Physique - Matière et Rayonnement, 4 place Jussieu, F-75252 Paris cedex 05, France; 3 Elettra-Sincrotrone Trieste, SS 14-km 163.5, I-34149 Basovizza, Trieste, Italy; 4 Physique des Interactions Ioniques et Moléculaires, Université Aix-Marseille, CNRS UMR 7345, Avenue Escadrille Normandie-Niémen, F-13397 Marseille cedex 20, France

## Abstract

Stimulated emission is a fundamental process in nature that deserves to be investigated and understood in the extreme ultra-violet (EUV) and x-ray regimes. Today, this is definitely possible through high energy density free electron laser (FEL) beams. In this context, we give evidence for soft-x-ray stimulated emission from a magnesium oxide solid target pumped by EUV FEL pulses formed in the regime of travelling-wave amplified spontaneous emission in backward geometry. Our results combine two effects separately reported in previous works: emission in a privileged direction and existence of a material-dependent threshold for the stimulated emission. We develop a novel theoretical framework, based on coupled rate and transport equations taking into account the solid-density plasma state of the target. Our model accounts for both observed mechanisms that are the privileged direction for the stimulated emission of the Mg *L_2,3_* characteristic emission and the pumping threshold.

## INTRODUCTION

Since the pioneering work of Einstein, it is established that stimulated emission is difficult to trigger as soon as the energy of the stimulated photon increases, so that realization of an x-ray laser remains a hard task. Most of the available x-ray lasers use highly ionized plasma created in a capillary discharge or from a solid slab hit by an optical pulse as active media.^1^ The advent of x-ray free electron lasers (FELs) has paved the way for the observation of x-ray stimulated emission pumped by hard and soft x-ray pulses at the femtosecond time scale.

Recently, Rohringer *et al.* have demonstrated stimulated emission from a rare gas in a transmission geometry.^2^ Saturated stimulated emission has also been observed for a silicon solid target by Beye *et al.*,^3^ while Yoneda *et al.*^4^ reported a hard-x-ray inner-shell atomic laser with a copper target, both pumped by FEL pulses. Yoneda *et al.,*^4^ in a transmission geometry, detected in the dependence of the output energy versus the pump pulse energy a nonlinear enhancement from a pumping threshold, typical of lasing based on amplified stimulated emission (ASE). In the work of Beye *et al.,*^3^ taking place in the extreme ultraviolet (EUV) range in a backward geometry, the stimulated emission was enhanced in a privileged direction given by the balance between the absorption length of the pumping radiation and the interaction length of the emitted stimulated radiation; the saturation effect was evidenced, but no non-linear enhancement similar to the one of Ref. 4 was reported.

We present an experiment in the backward geometry with a magnesium oxide (MgO) target excited by extreme ultra-violet (EUV) FEL pulses. We have observed both effects separately noticed in Refs. 3 and 4, stimulating us to develop a novel theoretical framework capable of predicting the phenomenology of the stimulated x-ray emission from condensed materials. Indeed our model, based on rate and transport equations including the solid-density plasma state of the target, accounts for both observed mechanisms that are the privileged direction for the stimulated emission of the Mg *L_2,3_* characteristic emission (3sd-2p electron transition) as reported in Ref. 3 and the pumping threshold as observed in Ref. 4. The presented theoretical framework provides the basis for the development of novel coherent pulsed EUV and x-ray sources characterized by negligible spectral jitter and unprecedented intensity.

## EXPERIMENTAL DETAILS

The experiment was conducted at the Elastic and Inelastic Scattering–TIMEX beamline^5^ at the FERMI@Elettra facility operating in the FEL-1 mode. The 56.8 eV (21.8 nm) *s*-polarised exciting radiation corresponds to the 12th harmonic of the seed laser. Its bandwidth is 0.1 eV. Each pulse has a duration of about 65 fs (full width at half maximum, FWHM) and a mean energy of 95 *μ*J, which corresponds to approximately 10^13^ photons. The FEL beam intensity before the sample is monitored through a calibrated ionization chamber.^6^ The emitted radiation is recorded by using an avalanche photodiode (APD, Laser Components SAR1500x) detector with a slit width *w* of 1.0 mm positioned at a distance *D* = 120 mm away from the sample on a circular rotating ring. A (Al 40 nm/Mg 0.8 *μ*m/Al 40 nm) filter provided by Luxel is placed in front of the APD to reject the long wavelength radiations (visible, seeding laser) and the FEL exciting radiation but allowing transmission of the EUV Mg *L*_2,3_ emission with a rejection rate of 5 × 10^4^. The FEL beam can be focused on the sample at normal incidence. For a given detection angle, hundreds of single-shots are carried out on different neighboring places of the sample. The MgO target sample is a single crystal supplied by Neyco; the sample was polished with a 0.8 nm residual *rms* surface roughness. The crystal is cut along the (100) plane whose reticular distance is around 0.2 nm. So Bragg scattering (diffraction) of the incident (21.8 nm) or emitted wavelength (27.9 nm) is not possible by this crystal (λ/2d > 1).

## RESULTS

**FIG. 1.**
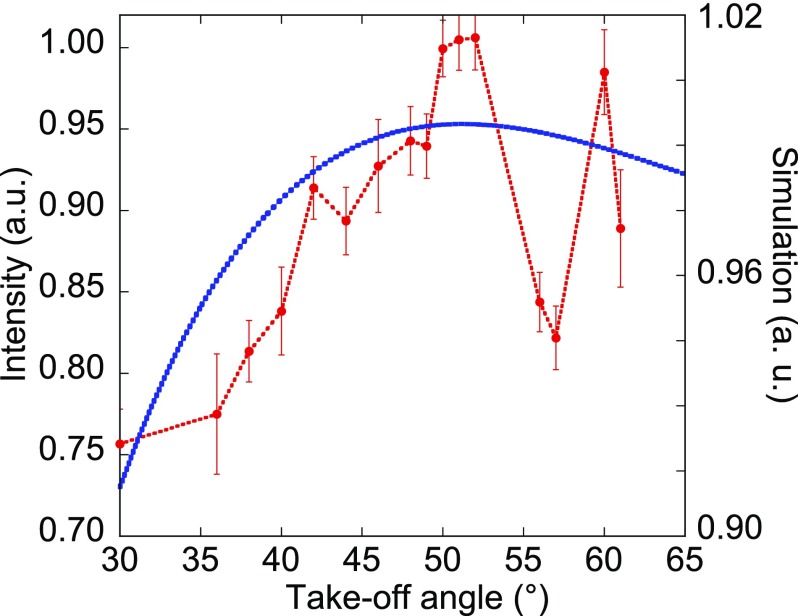
Angular distribution of the Mg *L_2,3_* emission generated in MgO upon the FEL irradiation at 56.8 eV: (points and thin dotted line) measurements from the avalanche photodiode detector; the error bars correspond to 3 statistical errors; (thick dotted line) simulation from the presented model.

The intensity of the emitted radiation is recorded as a function of the take-off or detection angle β as shown in Fig. 1. Each point corresponds to the mean of the measurements following hundreds of FEL shots, where each measurement is normalized by the energy in the FEL shot. The curve presents a broad asymmetric peak, with a maximum located around 50°. The errors bars represent 3 standard errors. Figure 1 also displays the angular distribution calculated by means of our theoretical model (see below). The experimental distribution displays some modulations which are likely actual structures considering the statistics of the measurements. Our model does not reproduce these oscillations. An explanation should be that the outgoing radiation is partially backscattered at the interface between the target and vacuum, resulting in interferences between the direct and backscattered radiations giving rise to these structures.

**FIG. 2.**
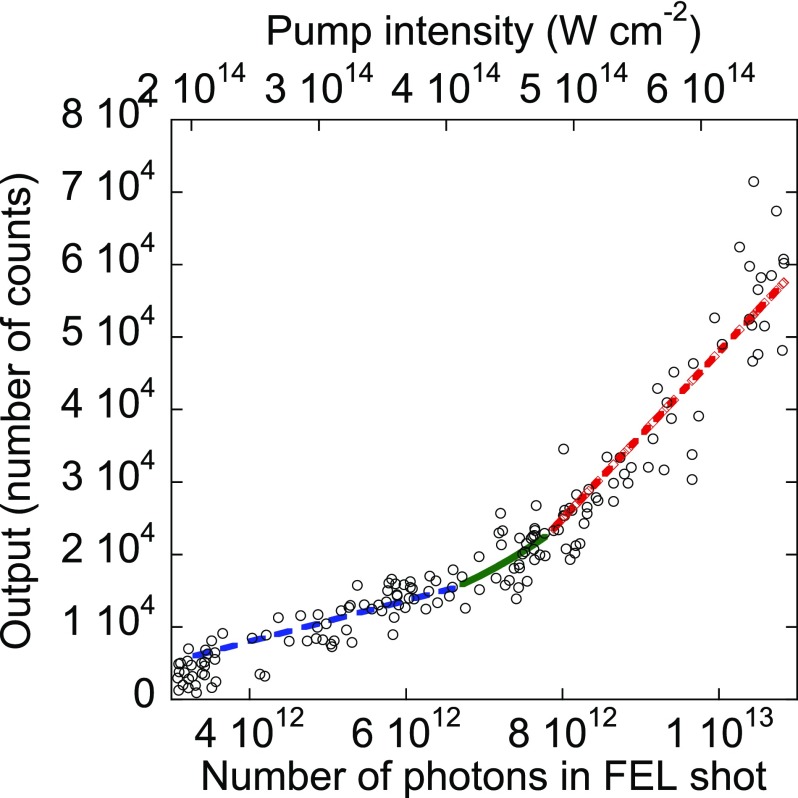
Number of characteristic photons detected by the avalanche photodiode, as a function of the number of photons in a FEL shot and of the pump intensity: (points) experimental values; (blue dashed line) region of the slightly increasing plateau; (red dashed line) linear fit according to Eq. (19); (green solid curve) transition zone between below and above threshold calculated from parametrized Eqs. (A9) and (A10). The energy of the photons in the FEL beam is 56.8 eV. The measurement is done with a take-off angle of 52°, close to the maximum of the angular distribution of the emitted radiation.

We also measured the output intensity of the generated emission as a function of the pump intensity or the number of photons in a FEL shot, Fig. 2. The measurement was done at a take-off angle of 52°, near the maximum of the angular distribution of the radiation. We observe, as the pump intensity increases, first a slowly increasing plateau up to a threshold value of about 7 × 10^12^ FEL photons/shot (4.3 × 10^14^ W cm^−2^) and then a large enhancement from this threshold value. The result of the simulation (see below) is also shown in this figure. As mentioned in Refs. 2 and 4 and explained in more detail below, this behaviour is typical of the travelling wave ASE^7^ with a clamping of the gain at the pumping threshold. The large intensity increase observed above the threshold cannot be ascribed to the generation of satellite lines following the creation of double core holes.^8,9^

## STATE OF THE SAMPLE UNDER FEL EXPOSURE

Under low intensity exposure, the MgO sample remains in a cold solid state and emits the Mg *L*_2,3_ band, different in MgO and metallic Mg. In the oxide, the spectrum presents two maxima located at 41 and 44.5 eV while the spectrum of metallic Mg forms a large band having its maximum around 49 eV.^10^ The spectral shift with respect to the metal comes from the insulating character of the oxide, leading to the existence of a forbidden band gap.^11,12^ Let us emphasize that stimulated emission in the x-ray domain from a crystalline solid differs notably from the two or three atomic level schemes implemented for lasers in the long wavelength domain. Electron transitions involving valence and conduction bands for the three relevant processes are as follows:

• ionization by soft x-ray FEL pulse: a core hole is created in a deep level of the atom by photo-ionization;^13^
• spontaneous emission: an electron from the valence band fulfils the core hole;
• stimulated emission: a spontaneous or a stimulated photon induces a stimulated emission with the creation of a new stimulated photon.

Core holes decay by both spontaneous and stimulated emissions. In competition with these two radiative decay channels, the Auger effect also reduces significantly the lifetime of the core holes. Nevertheless, core holes decay by stimulated emission more efficiently than by the Auger effect,^3^ so that under intense FEL pumping, the number of Auger processes is considerably reduced with respect to the small excitation regime (excitation with x-ray tube or synchrotron for instance) for which stimulation is irrelevant.

**FIG. 3.**
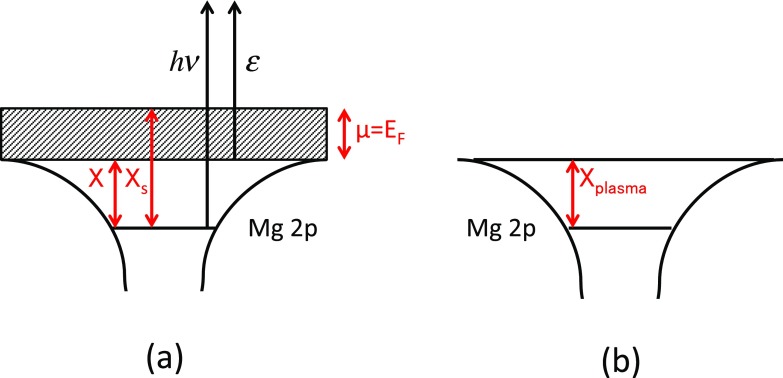
Energetic diagram of a solid in the cold state (a) and in the state of a warm plasma with solid density (b). In the cold solid state, the less tightly bound electrons are distributed within a valence band [dashed surface in (a)], whereas in the warm state, the electrons are distributed at discrete levels [horizontal lines in (b)].

In the cold solid, the ionization potential $X_{s}$ corresponds to the energy necessary to bring Mg 2p electrons above the Fermi energy $E_{F},\;$ see Fig. 3(a): *μ* is equal to the Fermi energy in the cold solid. The potential X corresponds merely to the bottom of the valence band. Under more intense exposure (typically 10^14^ W cm^−2^), the state of the target evolves towards the one of a warm plasma with solid density. The electronic band structure typical of the crystalline solid state tends to disappear as shown in Fig. 3(b). Somehow, X is the 2p ionization potential of the isolated atom decreased by the density-induced continuum lowering effect.

**FIG. 4.**
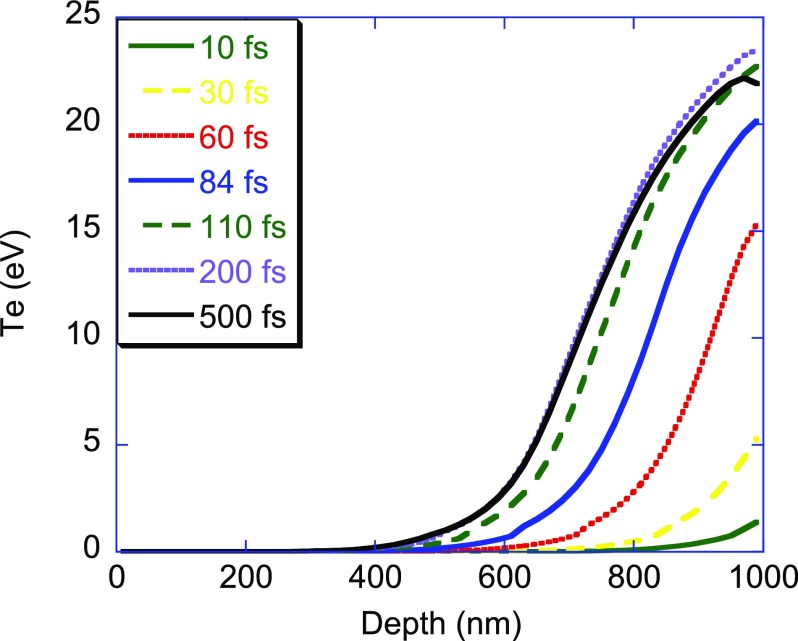
Depth variation of the electron temperature inside a Mg sample as a function time for the FEL pulse of 65 fs duration whose photons have an energy of 56.8 eV. The FEL beam arrives from the right at the depth of 1000 nm corresponding to the sample surface. The maximum of the pulse occurs at 84 fs.

At temperatures corresponding to the solid-density plasma and for times less than about 1 ps, the ionic lattice remains weakly altered but the electronic distribution can no longer be described by the density of states (DOS) of a cold crystalline solid. As shown in Fig. 4 for Mg metal upon irradiation pulses of 5 × 10^14^ W cm^−2^, the electron temperature Te obtained from the theoretical model given in Refs. 14 and 15 can increase up to 20 eV inside the sample. Let us note that owing to the difference between the densities of Mg and MgO, the number of magnesium atoms per volume unit is similar in both materials. In the simulation, we consider a degenerated free-electron gas and not the cold valence DOS which is supposed to disappear quickly as the electronic temperature increases.

The electron distribution obeys a modified Fermi-Dirac statistics (FDS). The change in the FDS creates free space below the Fermi energy and allows ionization at lower energy, see Fig. 3(b). In the heated solid, the potential X is always the ionization potential of the ion in the solid-density plasma and can be fixed by

$$X_{s}=\;X+\;E_{F}.$$ (1)

Since the ionization potential of the solids is very well known,^16^ it is easily possible to deduce X from Eq. (1). This shift is then handled with the use of Pauli-blocking factor

$$P\left(\varepsilon ,Te\right)=\;(1-F\left(\varepsilon ,Te\right)),$$ (2)

where $F\left(\varepsilon ,T\right)$ stands for the FDS and *k* is the Boltzmann constant

$$F\left(\varepsilon ,Te\right)=\;\frac{1}{1+\text{exp}\;\left(\frac{\varepsilon -\mu }{k\;Te}\right)}.$$ (3)

The energy ε  is obtained as the difference between the photon energy hν, where ν is the FEL carrier frequency, and the ionization potential X: ε=hν−X. At Te=0, the blocking factor is equal to zero and there is no transfer of electrons under $E_{F}$. At intermediate temperatures, the factor blocks only partially the transfer of electrons and the threshold extends between $X_{s}$ and $X_{\text{plasma}}$, and at high temperature, the factor has no effects leaving the potential at $X_{\text{plasma}}.$^15^ The chemical potential *μ* is obtained from the normalisation condition:

$$\int_{0}^{\infty }f\left(\varepsilon \right)\;F\left(\varepsilon ,Te\right)\;d\varepsilon =1.$$ (4)

Here, $f\left(\varepsilon \right)$ is the DOS in the valence band. The DOS evolves rather quickly with the temperature, so it is the same for the chemical potential, which can be approximated by the DOS of a Fermi free-electron gas

$$f\left(\varepsilon \right)=\;\frac{2}{{\left(2\;\pi \right)}^{2}}{\left(\frac{2m}{\hbar^{2}}\right)}^{3/2}\frac{1}{n_{e}}\;\sqrt{\varepsilon },$$ (5)

where $n_{e}$ is the electronic density, ℏ the reduced Planck constant, and *m* the electron mass. In our case for magnesium, one has numerically

$$n_{e}=8.61\times \;10^{22}\;{\text{cm}}^{-3},$$

$$f\left(\varepsilon \right)=4.2514{\times 10}^{33}\;\frac{1}{n_{e}}\;\sqrt{\varepsilon }\;in\;{\text{erg}}^{-1}\;\text{with}\;n_{e}\;in\;{\text{cm}}^{-3}\;\text{and}\;\varepsilon \;in\;eV,$$

kTe=0.03 eV,

$$\mu =\;E_{F}=7.1\;eV\;@\;kTe,$$

$$X=56.8-\;E_{F}\;in\;eV.$$

## RATE AND TRANSPORT EQUATIONS

The density of core holes $\rho_{c}\left(P,t\right)\;$ generated by the incident photon of energy hυ at a point ***P*** is governed by the rate equation

$$\partial_{t}\;\rho_{c}\left(P,t\right)=\;p\;\left(N\left(P\right)-\rho_{c}\left(P,\;t\right)\right)-\;\frac{\rho_{c}\left(P,\;t\right)}{\tau_{c}}-\;r{\;\rho }_{c}\left(P,t\right),$$ (6)

where *N(****P****)* is the concentration of atoms. The first term p, which is a source term, is the photoionization rate including the Pauli-blocking factor $P\left(\varepsilon ,T\right)$,

$${p=\;\sigma }_{ion}\;\frac{\phi \left(P,t\right)\;}{h\nu }P\left(\varepsilon ,T\right),$$ (7)

where $\phi \left(P,t\right)$ is the local FEL intensity and $\sigma_{ion}$ is the photoionization cross-section quantifying the creation of core holes. The second term accounts for the decrease in the core hole by the introduction of a phenomenological lifetime $\tau_{c}$, which may include possible collisional ionization effects. The last term *r* is the stimulated recombination rate from the valence band given by

$${r=n_{e}\;\sigma }_{sti}\;\frac{I_{\Sigma }\left(P,t\right)}{h\nu }\;\sqrt{\frac{2\;\varepsilon \;}{m}}\;h\;f\left(\varepsilon \right)\;F\left(\varepsilon ,T\right).$$ (8)

Here, $\sigma_{sti}$ is the cross-section for the stimulated effect and $I_{\Sigma }\left(P,t\right)$ the spontaneous and stimulated intensities coming at P from the whole volume Σ. The cross-section ${\;\sigma }_{sti}$ is related to ${\;\sigma }_{ion}$ by a micro-reversibility relationship (Einstein-Milne equation)

$${\;\sigma }_{sti}=\;{\;\sigma }_{ion}\;\frac{h^{2}}{16\;\pi \;m\;}\;R\;\frac{1}{\varepsilon },$$ (9)

where $R=\frac{g_{↓}}{g_{↑}}$ is the ratio of the statistical weight of the lower and upper levels of the transition. Let us outline that ${\;\sigma }_{sti}$ has the dimension of a square surface (cm^4^) since the process involves one photon and one electron. In our case,

$${\;\sigma }_{sti}=\;5.9846\times \;10^{-16}{\;\sigma }_{ion}\;R\;\frac{1}{\varepsilon }\;(in\;{\text{cm}}^{4})$$

with ${\;\sigma }_{ion}$ in cm^2^, ε in eV, and R=1/6 for a core hole in the subshell 2p of Mg. The lifetime $\tau_{c}$ is estimated to be 11 fs. A discussion concerning the micro-reversibility relations can be found in various textbooks.^17,18^

**FIG. 5.**
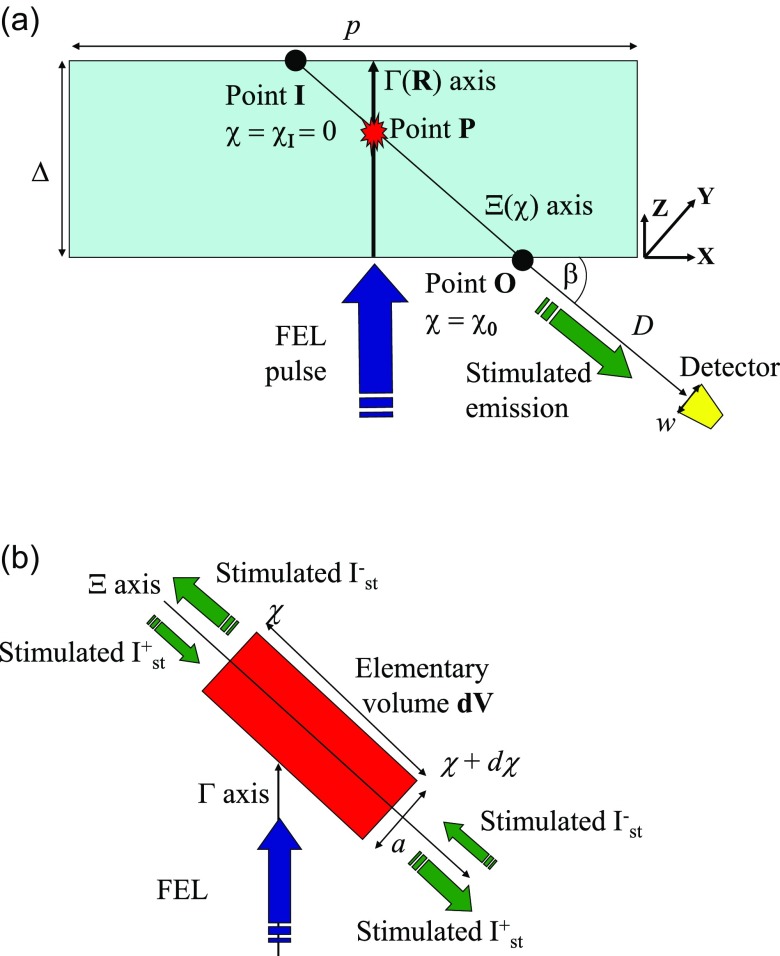
Geometry of the experiment. (a) View of the experimental geometry; the stimulated formation elementary volume ***dV*** is shown as a red star. The line Γ corresponds to the creation of core holes along the incident direction of the FEL radiation; the line Ξ is an interaction stripe in the direction β. (b) Zoom of the stimulated formation elementary volume ***dV*** at the point **P** along the line Ξ at the take-off angle β.

The initial condition, meaning that no core holes are present before the arrival of the FEL pulse

$$\rho_{c}\left(P,0\right)=0,\;\forall P\;\in \;\Sigma ,$$ (10)

must also be satisfied, where Σ stands for the domain of the ***P*** points (see Fig. 5). Let us precise that Σ corresponds to the interaction volume of the sample in which the stimulated radiation is created and can escape the target, which is in the order of the surface of the pulse footprint for the lateral dimension $({∼p}^{2})\;$ and in the order of the penetration depth Δ of the FEL pulse, Fig. 5. The value of *p* is 15 *μ*m and the one of Δ is a few tens of nm, the attenuation length at the FEL energy being 22 nm. An accurate calculation of the quantity $I_{\Sigma }\left(P,t\right)$ is a difficult task: one has to calculate the intensity at the point P and each time coming from each point of the whole volume Σ including the amplification and absorption along all paths and reflections on the boundary. In fact, we adopt a simplified quasi-analytical model where a volume integral (3D) is reduced to a one-dimensional problem (see Methods).

From Eqs. (1)–(10) and the spatio-temporal profile of the FEL pulse by a Gaussian function, it is possible to calculate the density of core holes at a point ***P*** by setting $t\equiv \;t_{P}=\frac{R_{P}}{v}$, with v being the velocity of the FEL pulse within the domain Σ and $R_{P}$ the distance between the surface and the point ***P***. The stimulated emission, which is detected in the direction given by the angle β, is generated from spontaneous emission at a point P along an axis Γ (in the domain Σ) where core holes are created by the ionizing incident FEL radiation, Fig. 5(a). Then, the spontaneous and stimulated emission grows along a stripe forming an interaction region on the line **Ξ,** being seeded from the spontaneous and stimulated radiations, but is also attenuated, mainly by photo-absorption along the interaction region. In 3D, this stripe can be regarded as a tube of diameter *a* and length $\chi_{O}-\chi_{P}$ that is the distance between the point P and the point O where the emission exits the sample, see Fig. 5(a). This geometry presents a close analogy with the pencil-like geometry adopted in some models of amplification of spontaneous emission ASE with transverse pumping.^7^

In this geometry, Fig. 5(b), the total number of emitted photons $I\left(\chi \right)$ generated along the axis **Ξ** in the elementary volume $dV=\frac{\pi }{4}\;a^{2}\;d\chi$ corresponding to the interval [χ,χ* *+ *d*χ] is given by differential equations taking into account the source term, the amplification by stimulation, the loss mainly by photo-absorption, and the geometry. In this elementary volume dV, two counter-propagating beams propagate, one along the +χ direction with an intensity $I^{+}\left(\chi \right)$ and the other along the −χ direction with an intensity $I^{-}\left(\chi \right)$, the total intensity $I\left(\chi \right)$ being $I^{+}\left(\chi \right)$ + $I^{-}\left(\chi \right)$. The set of coupled differential equations governing the growth in intensity reads

$${\pm \partial }_{\chi }\;I^{\pm }\left(\chi \right)\;=\rho_{c}\left(\chi \right)\;\sigma_{st}\;\frac{I^{\pm }\left(\chi \right)}{1+s\;I\left(\chi \right)}+\frac{F^{\pm }\left(\chi \right)}{T}\;\rho_{c}\left(\chi \right)\;-\;\frac{I^{\pm }\left(\chi \right)}{L},$$ (11)

which can be rewritten for convenience

$${\pm \partial }_{\chi }\;I^{\pm }\left(\chi \right)\;=\left(G\left(\chi \right)-\alpha \right)\;I^{\pm }\left(\chi \right)+\frac{F^{\pm }\left(\chi \right)}{T}\;\rho_{c}\left(\chi \right)$$ (12)

where $G\left(\chi \right)=\frac{\rho_{c}\left(\chi \right)\;\sigma_{st\;}}{1+s\;I\left(\chi \right)},\;\alpha =\frac{1}{L}$, and *σ_st_* (in cm^2^) is the stimulation cross-section. Also, *ρ_c_σ_st_* stands for the coefficient of stimulated emission and it is such that *ρ_c_σ_st_I* = *ρ_c_rhν*. This formula, where *r* is given by Eq. (8), is used to calculate *σ_st_* from *σ_sti_*.

On the right side of Eq. (11), the first term takes into account the saturation effect, the second one is a source term associated with the spontaneous emission, and the last one describes the attenuation (loss term mainly by photo-absorption). The saturation parameter *s*, inverse of the saturation intensity, is equal to $\sigma_{st}\tau_{c}$, T is a time constant governing the kinetics, and L is the absorption length given by

$$L=\;\frac{\Lambda }{\text{sin}\beta },$$ (13)

where Λ is the length over which the stimulated radiation intensity would be perpendicularly attenuated by the factor *e*^−1^. Following Ref. 3, the time T is calculated from the second central moment of $\rho_{c}\left(t\right)\;$ for temporal Gaussian pulse of FWHM duration τ,

$$T=\;\sqrt{\tau^{2}+{\tau_{c}}^{2}}.$$ (14)

The terms $F^{\pm }\left(\chi \right)$ are proportional to the spontaneous fluorescence yield $\omega_{sp}$ and to geometrical factors $g^{\pm }\left(\chi \right)$:

$$F^{\pm }\left(\chi \right)=\omega_{sp}\;g^{\pm }\left(\chi \right).$$ (15)

The factors $g^{\pm }\left(\chi \right)$ correspond to the solid angle into which spontaneously emitted photons may be radiated and so contribute to the output radiation

$$g^{+}\left(\chi \right)=\;\frac{1}{2}\;\left(1-\;\frac{\chi_{0}-\chi \;}{\sqrt{{\left(\chi_{0}-\chi \right)}^{2}+\;\frac{a^{2}}{4}}}\right);\;g^{-}\left(\chi \right)=\;\frac{1}{2}\;\left(1-\;\frac{\chi \;}{\sqrt{\chi^{2}+\;\frac{a^{2}}{4}}}\right).$$ (16)

The number of photons $I_{D}\;$ formed along the pumped pencil-like tube from the point P (with $\chi =\chi_{P}$), up to the point O (with $\chi =\chi_{O}$), exiting the domain Σ in the direction given by the take-off angle β and reaching the detector is given by the integral $\int_{\chi_{P}}^{\chi_{O}}I^{+}\left(\chi \right)\;d\chi$ with the assumption that the quantity $F^{+}$ is independent of χ since the distance to the detector D is large with respect to $\chi_{O}\;$ so that

$$F^{+}≅F_{D}\equiv \;\frac{\omega_{sp}}{2}\;\left(1-\;\frac{D\;}{\sqrt{D^{2}+\frac{w^{2}}{4}}}\right).$$ (17)

The rate and transport equations, Eqs. (6) and (11), respectively, form a set of coupled differential equations numerically solved by using the method of first-order finite difference with the following boundary conditions, Fig. 5(a):

$$\begin{array}{cc} I^{+}\left(\chi_{I}\right)=0 & \text{and}\;I^{-}\left(\chi_{O}\right)=0 \end{array}.$$ (18)

The computation is carried out by means of the refractive index values from the Centre for X-Ray Optics database.^19^ The different parameters used in the calculation are collated in Table I. For high intensities of the exciting FEL pulse, an absorption saturation effect occurs. This effect has been incorporated in the model by assuming that the imaginary part of $n\left(\omega \right)$ can be modeled by a linear dependence of the absorption coefficient on the energy density deposited in the target volume.^20^ Since no information is available for Mg in oxide, we have taken the value corresponding to the metallic state.

**TABLE I.** Physical quantities and experimental parameters used in the model for the MgO target. Values without reference are experimental parameters or calculated with our model.

Real part of *n* @ FEL carrier frequency (Ref. 19)	0.97
Imaginary part of *n* @ FEL the carrier frequency (Ref. 19)	8 × 10^−2^
Attenuation length Λ @ stimulated emission frequency (Ref. 19)	29 nm
Ionisation cross-section $\sigma_{ion}$ (Ref. 19)	3.4 × 10^−4^ nm^2^
Stimulation cross-section $\sigma_{st}\;$	0.56 × 10^−4^ nm^2^
Estimated FWHM pulse duration τ	65 fs
Core hole lifetime $\tau_{c}$ (Ref. 21)	11 fs
Mg atom density	49 nm^−3^
Lateral FEL beam size p	15 × 10^3^ nm
Fluorescence yield $\omega_{sp}$ (Ref. 22)	5.5 × 10^−4^
Saturation flux (saturation intensity)	9 × 10^30^ ph s^−1^ cm^−2^ (0.7 × 10^14^ W cm^−2^)

## DISCUSSION

The experimental pumping threshold value is in a fair agreement with the theoretical value $\phi_{th}\;$ 5.14 × 10^14^ W cm^−2^, see Eq. (A13). Above threshold, both core hole density and gain become clamped near their threshold values and the stimulated intensity varies linearly with the exciting photon intensity

$$\frac{\eta \;\left(N-\;{\rho_{c}}_{th}\right){\;\sigma }_{ion\;}}{G_{th}\;}\;(\phi -\;\phi_{th})$$ (19)

as shown by the red line in Fig. 2, where η stands for the detection efficiency taking into account the geometry (solid angle) and the combination of the APD efficiency and the filter transmittance, and ${\rho_{c}}_{th}$, $G_{th},$ and $\phi_{th}$ are the core hole density, the gain, and the FEL photon flux at threshold, respectively. The clamping can be understood by defining a lifetime of a stimulated core hole $\tau_{st}$,

$$\tau_{st}=\;\frac{\rho_{c}}{I^{tot}\;G\;v_{g\;}\;}.$$ (20)

It appears that the inverse dependence of the core hole stimulated lifetime on $I^{tot}$ corresponds to a negative feedback preventing $\rho_{c}\;$ from going beyond its threshold value. The pump intensity at the beginning of the plateau (2.15 × 10^14^ W cm^−2^) is slightly larger than the calculated saturation intensity 0.7 × 10^14^ W cm^−2^. The model allows us to reproduce the general shape of the angular distribution of the stimulated radiation for this experiment with MgO (and also for the experiment reported with Si by Beye *et al.*^3^) Nevertheless, the experimental distributions display some modulations which are likely actual structures considering the statistics of the measurements. Our model does not reproduce these oscillations. An explanation should be that the outgoing radiation is partially backscattered at the interface between the target and vacuum resulting in interferences between the direct and backscattered radiations giving rise to these structures.

In the presented experimental schemes, no optical feedback is delivered, so that the amplification of the stimulated emission is limited. A mean to circumvent this point is to make a distributed feedback (DFB) laser, i.e., a laser in which the active medium is also the optical medium necessary for the feedback. Owing to the previous works on Si^3^ and Cu^4^ and this one on MgO, it seems now possible to achieve DFB lasers with periodic nanometer multilayers^23^ in the EUV and soft x-ray ranges, and with crystals in the soft and hard x-ray ranges.^24,25^

## APPENDIX: CALCUALTION DETAILS

### Calculation of I_Σ_

An expression for $I_{\Sigma }$ can be obtained by determining the photon flux density ${dI}_{dv}\;$ from the elementary volume dv and then integrating over the volume Σ.  Taking into account the isotropic character of the emission and assuming a steady-state and uniform situation ($\rho_{c}$ independent of the position and time), one has

$${dI}_{dv}\;=E\;\frac{\rho_{c}{\;\sigma }_{tot}\;dv}{\tau_{rad}\;4\;\pi^{2}\;l^{2}},$$ (A1)

where ${\;\sigma }_{tot}$ is the total cross-section for spontaneous and stimulated emission, $\tau_{rad}$ the radiative lifetime for spontaneous emission, and l the distance between the emitting elementary volume dv and the point P. Since the emitted photons experience a gain coefficient *g* along their path L,  the rate of photons seen at P is given by

$${dI}_{dv}\left(P\right)=\;{dI}_{dv}\;\;\text{exp}\;\left(\int_{L}\left(G-\alpha \right)\;dL\right)$$ (A2)

and the total flux $I_{\Sigma }\left(P\right)\;$ from Σ seen at P is given by

$$I_{\Sigma }\left(P\right)\;=\frac{\;\rho_{c}{\;\sigma }_{tot}}{\tau_{rad}\;4\;\pi^{2}}{∭}_{\Sigma }\frac{1\;}{\;l^{2}}\;dv\;\;\;\text{exp}\;\left(\int_{L}\left(G-\alpha \right)\;dL\right).$$ (A3)

An accurate (numerical) calculation of this quantity is very costly in terms of computation time so that some approximations have been done. Our goal is to estimate the on-axis Ξ effects of transverse excitations and then the calculation is performed along the line Ξ at each position χ of interest so that a cylindrical reference system (ρ,χ) has been implemented. In this system, $I_{\Sigma }\left(P\right)$ can be rewritten as

$$I_{\Sigma }\left(\chi_{P}\right)=\frac{\;\rho_{c}{\;\sigma }_{tot}}{\tau_{rad}\;4\;\pi^{2}}\;\int_{0}^{L\;}G\left(\chi \right)d\chi \int_{0}^{R}\rho \;d\rho \;\frac{1}{2\;[\rho^{2}+{\left(\chi -\chi_{P}\right)}^{2}]}\;\text{exp}\left(\frac{\sqrt{\left[\rho^{2}+{\left(\chi -\chi_{P}\right)}^{2}\right]}}{\chi -\chi_{P}}\int_{\chi_{P}}^{\chi }(G(\chi^{\prime })-\alpha )\;d\chi^{\prime }\right),$$ (A4)

where R is the radius of the cylinder of axis Ξ totally included inside the sample.

Setting $t^{2}\left(\rho \right)=\rho^{2}+{\left(\chi -\chi_{P}\right)}^{2}$ and $s\left(\chi ,\chi_{P}\right)=\frac{\int_{\chi_{P}}^{\chi }(G(\chi^{\prime })-\alpha )\;d\chi^{\prime }}{\chi -\chi_{P}}$ gives for the integral over ρ,

$$\int_{0}^{R}\rho \;d\rho \;\frac{1}{2\;\left[\rho^{2}+{\left(\chi -\chi_{P}\right)}^{2}\right]}\;\text{exp}\left(\frac{\sqrt{\left[\rho^{2}+{\left(\chi -\chi_{P}\right)}^{2}\right]}}{\chi -\chi_{P}}\int_{\chi_{P}}^{\chi }(G(\chi^{\prime })-\alpha )\;d\chi^{\prime }\;\right)=\int_{t(0)}^{t(R)}\frac{\text{exp}(s\left(\chi ,\chi_{P}\right)\;t)}{2\;t}\;dt=1/2{\left\{Ln\left[t\right]+\sum_{n=1}^{\infty }\frac{s\left(\chi ,\chi_{P}\right)\;t}{n\;n!}\;\right\}}_{t(0)}^{t(R)}.$$ (A5)

### Steady-state solution of the coupled system of rate and transport equations

Considering that the emitted radiation travels with a group velocity $v_{g\;}$ so that

$$\chi =\;v_{g\;}t,$$ (A6)

the transport equations, Eq. (11), become

$${\pm \partial }_{t}I^{\pm }≅\frac{G\;v_{g\;}I^{\pm }}{1+s\;I}\;-\;\frac{I^{\pm }\;}{\tau_{p}}+{\;R}_{sp}$$ (A7)

with

$$\tau_{p}=\frac{L}{v_{g\;}};{\;R}_{sp}=\;v_{g\;}\;\frac{F^{\pm }\;}{T}\rho_{c}.$$ (A8)

$\tau_{p}$ can be regarded as the photon lifetime along the interaction stripe and the term ${\;R}_{sp}$ corresponds to the spontaneous radiation source. Without saturation and in the steady state ($\partial_{t}I^{\pm }=0;\;\partial_{t}\rho_{c}=0;s\approx 0;G=\rho_{c}{\;\sigma }_{st\;}$), the radiated intensity is given by

$$I^{\pm }\;\left(\rho_{c}\right)=\frac{{\;R}_{sp}}{\frac{1\;}{\tau_{p}}-v_{g\;}G\;}$$ (A9)

and the steady-state incident FEL intensity reads, see Eq. (6),

$$\phi \;\left(\rho_{c}\right)=\frac{1}{\left(\;N-\;\rho_{c}\right)\sigma_{ion}}\;\left(\frac{\rho_{c}}{\tau_{c}}+G\;I\left(\rho_{c}\right)\;\right).$$ (A10)

When $\frac{1\;}{\tau_{p}}=v_{g\;}G$, it appears that the radiated intensity presents a catastrophic behaviour. If one defines the threshold gain $G_{th}$ by

$$G_{th}\;=\;\frac{1\;}{{v_{g\;}\tau }_{p}}=\;\frac{1}{L}$$ (A11)

then it corresponds a core hole density threshold ${\rho_{c}}_{th}=\rho_{c}(G_{th})$,

$${\rho_{c}}_{th}=\;\frac{1}{L\;\sigma_{st}\;}$$ (A12)

and a pumping threshold $\phi_{th}$,

$$\phi_{th}\;=\frac{1}{L\;\sigma_{st}\;}\;\frac{1}{\left(N-\;\frac{1}{L\;\sigma_{st}\;}\right){\sigma_{ion\;}\tau }_{c}}.$$ (A13)

Below threshold, the detected intensity $I_{D}$, mainly made of spontaneous radiation, whose intensity $I_{\text{spon}\;}$ is proportional to ϕ,  while the stimulated intensity $I_{st}$ is close to zero, is given by

$$I_{D}≅\;I_{\text{spon}\;}\;∼\;\phi ;\;I_{st}\;≅0.$$ (A14)

Above threshold, this intensity is formed with constant spontaneous radiation intensity and with stimulated radiation intensity, which varies linearly with the exciting photon flux

$$I_{D}≅\;I_{\text{spon}\;}+\;I_{st};\;I_{st}∼\;\phi -\phi_{th};\;I_{\text{spon}}\;∼\;\phi_{th}.$$ (A15)

The transition domain between below and above threshold can be conveniently described by considering Eqs. (A9) and (A10) as a set of parametrized equations, with $\rho_{c}$ being the free parameter of the system.
